# Epicardial Epithelial-to-Mesenchymal Transition in Heart Development and Disease

**DOI:** 10.3390/jcm5020027

**Published:** 2016-02-19

**Authors:** Michael Krainock, Omar Toubat, Soula Danopoulos, Allison Beckham, David Warburton, Richard Kim

**Affiliations:** Division of Cardiothoracic Surgery, University of Southern California, Los Angeles, CA 90027, USA; mkrainock@llu.edu (M.K.); toubat@usc.edu (O.T.); sdanopoulos@chla.usc.edu (S.D.); abeckham@chla.usc.edu (A.B.); dwarburton@chla.usc.edu (D.W.)

**Keywords:** epicardium, epithelial-to-mesenchymal transition, heart development

## Abstract

The epicardium is an epithelial monolayer that plays a central role in heart development and the myocardial response to injury. Recent developments in our understanding of epicardial cell biology have revealed this layer to be a dynamic participant in fundamental processes underlying the development of the embryonic ventricles, the coronary vasculature, and the cardiac valves. Likewise, recent data have identified the epicardium as an important contributor to reparative and regenerative processes in the injured myocardium. These essential functions of the epicardium rely on both non-cell autonomous and cell-autonomous mechanisms, with the latter featuring the process of epicardial Epithelial-to-Mesenchymal Transition (EMT). This review will focus on the induction and regulation of epicardial EMT, as it pertains to both cardiogenesis and the response of the myocardium to injury.

## 1. Introduction

The epicardium consists of an epithelial monolayer located on the outermost surface of the heart, serving as a boundary between the underlying myocardium and the pericardial cavity. Early developmental biologists regarded the epicardium as an inert barrier, and thus the functional importance of epicardial cells in both heart development and cardiac disease was largely neglected throughout much of the late 19th and early 20th centuries. We now know that the epicardium is a dynamic structure that actively participates in both cardiogenesis and the response of the post-natal myocardium to injury, via both non-cell autonomous and cell autonomous mechanisms.

**Figure 1:**
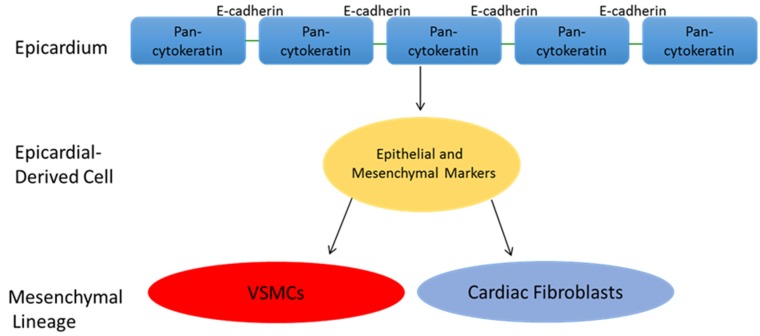
Epicardial EMT features the generation of Epicardial Derived Cells (EPDCs) that co-express both epithelial and mesenchymal markers. This multipotent intermediary cell then goes on to adopt a mesenchymal lineage. There is agreement that EPDCs differentiate into vascular smooth muscle cells (VSMCs) of the coronary vasculature, as well as interstitial and peri-vascular fibroblasts. However, the extent to which they differentiate into cardiomyocytes and endothelial cells, if they do at all, is controversial.

The non-cell autonomous function of the epicardium relies on the elaboration of paracrine factors that support myocardial growth, largely through the regulation of cardiomyocyte proliferation. In 2011, Li and colleagues demonstrated that insulin-like growth factor 2 (IGF2) secreted by the epicardium is a key regulator of ventricular cardiomyocyte proliferation during development. Global knockout of *Igf2* and conditional knockout of the IGF2 receptors *Igfl2* and *Insr* both resulted in reduced proliferaton and the development of hypoplastic ventricular chambers [1]. In 2013, Huang *et al*. illustrated that insulin-like growth factor 2b (Igf2b) is similiarly important for cardiomyocyte proliferation in the developing Zebrafish heart. Importantly, these authors further demonstrated that Igf2b is required for cardiomyocyte proliferation during myocardial regeneration following injury, particularly in a population of sub-epicardial cardiomyocytes marked by gata4:EGFP and thought to be central to the re-population of cardiomyocytes in the infarct zone [2]. Conversely, the cell-autonomous functions of the epicardium feature the direct participation of epicardial derived mesenchymal progenitor cells in cardiogenic processes that underlie coronary vasculogenesis, valvulogenesis, and the cellularization of the ventricles. These epicardial derived cells (EPDCs) are generated through the process of epicardial Epithelial-to-Mesenchymal Transition (epicardial EMT), by which epicardial cells delaminate from the surrounding monolayer to form a sub-epicardial mesenchyme, and eventually differentiate into cells of mesenchymal lineage. While the cell fate of EPDCs has been definitively demonstrated to include the fibroblast and vascular smooth muscle cell lineages, the extent to which EPDCs differentiate into cardiomyocytes and endothelial cells is a subject of debate (Figure 1). The following sections will focus on the formation of the epicardium, the induction and regulation of the epicardial EMT process, and the fate of EPDCs generated during heart development. Additionally, the role of epicardial EMT in the repair of the post-natally injured heart will be further discussed.

## 2. Formation of the Embryonic Epicardium

Early practitioners of cardiovascular embryology regarded the epicardium as an extension of the myocardial heart tube, serving simply as a barrier between the underlying myocardium and the pericardial cavity. Consequently, the role of the epicardium in cardiogenesis was long overlooked. This view was first questioned during the late 19th and early 20th centuries, owing to the observation that progenitor cells intrinsic to the myocardial tube consisted almost exclusively of cardiomyoblasts, thus prompting the search for an extra-cardiac source of the epicardium [3].

In the 100-plus years since a cardiogenic origin of the epicardium was initially questioned, it has been definitively established that the epicardium arises from a villous outgrowth located on the ventral sinus venosus, termed the Proepicardial Organ (PEO) [3]. This structure is evolutionarily conserved, with PEOs identified in humans and other mammals, fish, and birds [3,4,5,6]. Recent work has demonstrated that the PEO is a heterogenous structure with diverse embryologic origins, to include the embryonic mesothelium and the lateral plate mesoderm [7]. These diverse origins add a layer of complexity to the functional differentiation potential of the PEO, and much work is underway to establish exactly which cell lineages originate from distinct progenitor cell populations within the PEO, as disussed further below.

The earliest investigations of the PEO were carried out primarily in avian species. In the avian embryo, the PEO is first recognizable as a villous protrusion extending from the ventral limit of the sinus venosus at Hamburger-Hamilton stage 13/14 [3]. At HH17/19, cells from the PEO make contact with the heart and spread out over the myocardium, starting in the region of the atrioventricular grooves, and eventually extending distally to completely invest the ventricles by HH26/27 [8]. The PEO is a transient structure, and recent evidence suggests that it is composed of multiple separate progenitor sub-populations with distinct differentiation potentials. In 2012, Katz *et al*. demonstrated that a subset of cells within the PEO give rise to coronary vascular endothelial cells, as well as cells of the sinus venosus and endocardium. These cells specifically express the transcription factors Scleraxis and Semaphorin3D, and appear to be genetically distinct from their *Tbx18/Wt1* expressing counterparts within the PEO [9]. Additional work by Acharya and colleagues, as well as Kikuchi *et al*., demonstrated that PEO progenitor cells expessing *Tcf21* preferentially give rise to cells of the cardiac fibroblast lineage [10,11]. These data thus support a model in which genetic determination of fate specification occurs prior to epicardial EMT, and perhaps prior to the assembly of progenitor cells within the PEO. The PEO thus appears to serve as a primordial anlage with heterogenous embryologic origins, and distinct compartmentalization of progenitor cells with different lineage commitment potentials. Current efforts aim to better characterize the cellular composition of the PEO, and to shed light on fate decisions both prior to organization of the PEO, and within the PEO proper.

Regardless of when PEO cell fate is determined, cells from the PEO eventually migrate over the surface of the developing heart to form the epicardium. Shortly after epicardial investment has begun, a subset of epicardial cells overlying the AV groove separate from the epicardial monolayer and form the sub-epicardial mesenchyme. These cells, termed Epicardial Derived Cell (EPDCs), initially express both epithelial and mesenchymal markers, and represent a dedifferentiation of epithelial cells to an intermediary cell with enhanced developmental plasticity and intriguing multipotency [12].

## 3. Induction of Epicardial EMT

The epicardial EMT process begins in the area of the AV-grooves, and evidence suggests that this regional specificity is mediated at least in part by the spatially regulated elaboration of growth factors from the underlying myocardium. Both *in vitro* and *in vivo* experimental data have implicated a heterogeneous group of molecules in the induction of epicardial cell transformation.

The ability to culture epicardial explants *in vitro*, and more recently to perform epicardium-restricted genetic knockouts, has allowed for investigation into the effect of a variety of growth factors on epicardial EMT. When avian or murine embryonic hearts are cultured on glass or collagen, a monolayer of epicardial cells spreads out over the growth substrate. When kept in serum-free culture, these monolayers maintain their epithelial identity, thus readily lending themselves to experimentation with exogenously delivered substances.

In 2001, Morabito *et al*. demonstrated that the addition of FGFs 1,2, and 7, VEGF, EGF, and TGFβ1 all induced the mesenchymal transformation of avian epicardium grown on collagen gels [13]. Subsequent investigation of TGFβ1 and TGFβ2 ligands carried out by Compton *et al*. demonstrated a central role for the ALK5 receptor in the transformative process [14]. *In vivo*, the murine myocardium expresses TGFβ2 in a spatially restricted fashion, limited to the AV canal and outflow tract, where epicardial EMT is most prominent [15]. Additionally, in a 2008 study, Sridurongrit *et al*. demonstrated that the epicardial-specific deletion of *Alk5* resulted in defective muscularization of sub-epicardial coronaries, as well as the abnormal formation of intra-myocardial capillaries. Furthermore, these investigators demonstrated that epicardial cells defective for *Alk5* failed to undergo TGFβ-mediated EMT *in vitro* [16].

In addition to TGFβ-ALK5 mediated signaling, genetic knockout studies have additionally implicated TGFβ Receptors 2 and 3 in epicardial EMT. Deletion of *Tgfβr2* in the myocardium does not impair development of the heart, however deletion in both the myocardium and the epicardium results in impaired coronary vasculogenesis and poor myocardial growth [17,18]. Similarly, in 2011, Sanchez *et al*. demonstrated that homozygous deletion of *Tgfβr3* resulted in reduced epicardial proliferation and impairment of EPDC invasion. These results were re-demonstrated *in* *vitro*, with the additional finding that loss of TGFβR3 impaired not only TGFβ-mediated epicardial EMT, but also FGF2 and HMW-HA induced transformation [19].

As mentioned above, FGFs 1, 2, and 7 stimulate epicardial EMT *in vitro* [13]. In addition, in 2011, Vega-Hernandez *et al*. used lineage-restricted conditional knockout studies and in-situ hybridization to demonstrate that myocardial-derived FGF10 interacts with epicardial-expressed FGFR1 and FGFR2 to induce EPDC invasion of the compact myocardium. Knockout of *Fgf10* or *Fgfr1/2* resulted in reduced EDPC invasion of the myocardium, and a reduction of cardiac fibroblasts within the myocardium [20]. Additional work by Lavine and colleagues revealed a role for FGF9 signaling via FGFR1/2 in Sonic Hedgehog (SHH)-mediated coronary vasculogenesis. These authors demonstrated that epicardial derived FGF9 induces the expression of *Shh*, which in turn promotes myocardial expression of *Vegf* and *Ang2.* Antagonism of SHH signling in cultured hearts resulted in reduced coronary plexus formation, and *in vivo* interruption of the SHH signaling pathway resulted in less subepicardial mesenchyme and reduced vascular plexus formation. These results were recapitulated with conditional knockout of both *Fgf9* and *Fgfr1/2*, with exogenous SHH rescuing vascular plexus formation [21,22,23]. Taken together, these data suggest that FGF9 induces *Shh* expression via FGFR1/2 signaling pathways, and that SHH is in turn important for myocardial and subepicardial plexus formation, at least in part via the induction of *Vegf* and *Ang2* expression.

In addition to the TGFβ and FGF ligands discussed above, recent work has also implicated both the α and β isoforms of the Platelet Derived Growth Factor Receptor (PDGFR) in the induction of epicardial EMT. In a 2011 study, Smith *et al*. demonstrated that the epicardial-specific deletion of both *Pdgfr* α/β resulted in normal epicardial investment, but a perturbation in epicardial EMT and EPDC generation. Intriguingly, knockout of each isoform individually resulted in markedly different phenotypes. Isolated deletion of *Pdgfrα* resulted in a reduction in cardiac fibroblast generation, while knockout of *Pdgfr*β alone resulted instead in an impairment of vascular smooth muscle generation. Molecular investigation of dual *Pdgfrα*/β epicardial knockout mice revealed a reduction in the expression of *Sox9*, *Slug*, and *Snail* [24]. These transcription factors are important for the transcriptional regulation of epicardial EMT, as will be discussed in the following section.

In addition to growth factor mediated induction of epicardial EMT, mounting experimental evidence suggests the Wnt/β-catenin and Notch signaling pathways also regulate the transformation of epicardial cells. Epicardial specific deletion of β-catenin resulted in reduced EPDCs in the sub-epicardial mesenchyme [25]. Conditional knockout of β-catenin in the Proepicardial Organ does not affect epicardial cell proliferation or epicardial investment of the myocardium, however there is impairment of myocardial EPDC invasion and differentiation of EPDCs into vascular smooth muscle cells of the coronary arteries [26]. Additionally, Notch mediates EPDC differentiation into vascular smooth muscle cells via the transcription factor RBPJ, and inhibition of epicardial Notch signaling results in impaired maturation of the coronary arteries [27,28].

The above discussion highlights the importance of myocardial derived growth factors and epicardial Wnt/ β-catenin and Notch signaling in the induction of epicardial EMT, and the differentiation of EPDCs into cells of mesenchymal lineage. The procession of epicardial EMT induction to final mesenchymal differentiation relies on major changes in the transcriptome of the epicardial cells undergoing transformation. The next section will present an up-to-date review of the transcriptional control of epicardial EMT, with coverage of both the major transcriptional pathways regulating the sentinel events of epicardial EMT, and the more specific transcription factors associated with EPDC mesenchymal lineage selection.

## 4. Transcriptional Regulation of Epicardial EMT

The earliest events of epicardial EMT feature an alteration in cell morphology and the delamination of cells from the epicardial monolayer. Following the creation of a sub-epicardial mesenchyme, EPDCs expressing dual epithelial and mesenchymal lineage markers invade the underlying myocardium and envelope the sub-epicardial coronaries. This EPDC generation is in turn followed by lineage selection and definitive mesenchymal transformation. This complex process is regulated by a diverse milieu of transcription factors, and recent work by a number of investigators has provided novel information regarding the specific roles of individual transcription factors throughout the epicardial EMT process.

### 4.1. Wt1 and Tbx18

Wilms Tumor 1 (WT1) is a zinc-finger transcription factor, initially reported in the literature as a mutated tumor suppressor in the setting of Wilms’ Tumor. Later work revealed *Wt1* expression in both the PEO and epicardium, and subsequent investigations have identified WT1 as a central mediator of epicardial EMT [29,30,31,32] . In 1999, Moore and colleagues reported that *Wt1* knockout mice demonstrated a paucity of EPDCs in the sub-epicardial mesenchyme, and died in the setting of pericardial hemorrhage [33]. This work was expanded upon in 2011, when von Gise *et al*. demonstrated that *Wt1* knockout resulted in impaired EMT and a loss of EPDC myocardial invasion. These authors additionally demonstrated that *Wt1* knockout resulted in an epicardium-specific reduction in β-catenin, with down-regulation of the Wnt/ β-catenin target *Lef1* [25]. In addition to impaired epicardial Wnt signaling, Martinez-Estrada *et al*. demonstrated that the epicardial-specific deletion of *Wt1* resulted in the up-regulation of *E-cadherin* expression, with a reduction in the transcription factor SNAIL1 [34]. As will be discussed in detail later, SNAIL1 plays a key role in epicardial EMT via the binding and down-regulation of *E-cadherin*, allowing for disassembly of epicardial adherens junction [35,36]. *In vitro*, WT1 was demonstrated to bind directly to the *Snail1* promoter in FACS-sorted epicardial cells, implicating a direct relationship between WT1 and SNAIL1 in the epicardium [34].

The importance of Tbx18 expression in epicardial EMT is less clear. While *Tbx18* is expressed in the embryonic PEO and epicardium, knockout does not impair epicardial EMT [29,37,38,39]. This may be due to redundancy in *Tbx20* and *Tbx18*, as *Tbx20* is also expressed in the embryonic epicardium, and has been demonstrated to support the mesenchymal transformation of endocardial cells during valve development [29,40,41,42]. When isolated in culture, TBX18 induces the mesenchymal transformation of primary murine epicardial cells via the induction of *Slug* [43].

### 4.2. Snail and Slug

Recent evidence from multiple investigators has revealed a potential role for the zinc-finger transcription factor, SNAIL, in epicardial EMT, similar to the role it plays in cancer EMT [29,34,36,44,45]. SNAIL binds to the *E-cadherin* promoter and represses its expression, thus promoting the disassembly of epithelial adherens junctions and enhancing cell motility [35,36]. Importantly, the disassembly of epithelial adherens junctions frees β-catenin and NF-κβ from the cell membrane, allowing these factors to translocate to the nucleus where they promote mesenchymal gene expression [46]. In avian epicardium specifically, *Snail* over-expression is sufficient to induce epicardial EMT *in vitro*, and enhances EPDC invasion into the myocardium through the up-regulation of Matrix Metalloproteinase 15 *in vivo* [45].

As mentioned above, the transcription factor SLUG has been demonstrated to induce epicardial EMT under the direction of TBX18. Additionally, WT1 has been demonstrated to mediate this process, and loss of *Slug* expression *in vitro* suppresses epicardial EMT [43].

### 4.3. Tcf21 and Nfatc1

The *Tcf21* gene encodes a bHLH transcription factor that is expressed in epicardial, pericardial, and coronary artery progenitor cells, as well as in the epicardium itself [29,47,48]. As mentioned previously, in 2012, Acharya *et al*. used lineage-tracing analysis to demonstrate that *Tcf21* is expressed in a subset of epicardial cells that eventually transform into cardiac fibroblasts. Inducible knockout of *Tcf21* resulted in impairment of epicardial EMT and a specific reduction in EMT-derived fibroblasts. Interestingly, *Tcf21* expression was detectable in populations of epicardial cells prior to the onset of EMT. Taken together, these data suggest that *Tcf21* plays a role in the induction of EMT in a specific subset of epicardial cells destined to become cardiac fibroblasts [10]. That same year, Braitsch *et al*. provided additional support for a lineage-specific role of *Tcf21*, demonstrating that *Tcf21* knockout resulted in a reduction in cardiac fibroblasts, with an increase in smooth muscle cell differentiation within the sub-epicardial mesenchyme, and only a modest effect on global epicardial EMT [49].

*Nfatc1* encodes a calcineurin dependent transcription factor expressed in the embryonic PEO, epicardium, EPDCs, and endocardial cushions [29,50]. Experimental evidence provided by Combs, *et al.* suggests that *Nfact1* expression promotes the myocardial invasion of EPDCs via the induction of the extracellular matrix-remodeling enzyme Cathepsin K. Conditional knockout of *Nfatc1* results in impairment of EPDC invasion into the myocardium, as well as impairment of coronary artery penetration into the myocardium [50].

### 4.4. Twist1, Scleraxis, Sox9, and Hand2

TWIST1 is a bHLH transcription factor that has been demonstrated to support EMT during cancer progression, in some instances in concert with SNAIL [51]. In the heart, TWIST1 supports valve development via enhancement of endocardial cushion cell proliferation and motility. In the epicardium, *Twist1* induction is concurrent with the onset of epicardial EMT, and continued expression of *Twist1* has been demonstrated in the EPDCs of both mice and chick [29,39,52,53]. Lineage tracing analysis suggests that *Twist1* is expressed in epicardial cells that eventually differentiate into fibroblasts and vascular smooth muscle cells, in-line with the differentiation profile resulting from known inducers of epicardial EMT [7]. While these findings certainly suggest a role for TWIST1 in the process of epicardial cell transformation, direct evidence is lacking.

Scleraxis is expressed in both the murine PEO and epicardium. Intriguingly, only a subset of cells within the PEO express Scleraxis, and these cells do not demonstrate concurrent expression of *Wt1* or *Tbx18* [9,29] Lineage tracing analysis has provided evidence that in contrast to *Wt1* and *Tbx18* expressing cells, Scleraxis positive progenitors eventually differentiate into coronary vascular endothelial cells and even endocardium and cardiomyocytes [9].. These data frame Scleraxis as a marker of an independent progenitor pool present in the PEO, that eventually contributes to cell compartments that are completely distinct from traditional epicardial EMT lineages. However, knockout studies have revealed that Scleraxis deficient mice demonstrate dysmorphic features in the fibrous annulus of the atrio-ventricular canal as well as the valvular leaflets , two cardiac structures dependent on epicardial EMT. Thus, the role Scleraxis plays in epicardial EMT and EPDC differentiation is not completely clear [29,52,54,55,56,57].

SOX9 is an SRY family transcription factor previously implicated in endocardial cushion EMT and valve development [29,54]. In 2011, Smith *et al*. demonstrated that epicardial-specific deletion of the PDGF receptors resulted in impaired EMT and a reduction in *Sox9* gene expression. This impairment was subsequently rescued with the over-expression of *Sox9*, providing evidence that *Sox9* is also important for epicardial EMT [58]. Similarly, HAND2 induces the expression of *Pdgfrα*, and loss of *Hand2* results in a loss of epicardial integrity and impairment of epicardial EMT [59].

### 4.5. Retinoic Acid

Retinoic Acid (RA) appears to play a central role in the regulation of both cell autonomous and non-cell autonomous functions of the epicardium. Using an *in vitro* explant culture method that allows for the separation of the epicardium, myocardium, and endocardium, Stuckmann *et al*. demonstrated that Erythropoietin (EPO) and RA produced by the epicardium are necessary for cardiomyocyte proliferation [60]. *In vivo*, the epicardium is the sole layer within the developing heart competent to secrete Retinoic Acid, owing to its expression of the retinaldehyde-dehydrogenase 2 protein, a key mediator of RA synthesis [61]. In addition to serving as an endogenous source of RA, the epicardium secretes proliferation-inducing mitogens in response to RA binding to the retinoid X receptor α (RXRα) in epicardial cells themselves. These largely uncharacterized mitogens have been demonstrated to promote cardiomyocyte proliferation via activation of the PI3K and ERK signaling pathways [62,63]. The mitogenic effect of RA on the myocardium appears to be completely dependent on the epicardium, as epicardial-specific expression of mutated RXRα results in a thin myocardium with a decreased number of cardiomyocytes, while endocardial and myocardial-specific mutation of the RA receptor have no effect on heart development [64]. While RA inducible epicardial mitogens remain an area of active investigation, FGF9 has been identified as one such factor with a potent proliferative effect on cardiomyocytes, and reduced expression of *Fgf9* results in decreased cardiomyocyte proliferation and ventricular hypoplasia [65].

Retinoic acid is also a central component of core signaling pathways regulating epicardial EMT. WT1 directly regulates *Raldh2* expression in the epicardium, and RA supplementation partially rescues epicardial EMT in *Wt1* knockout hearts [25,66]. Additionally, RA exerts control over EPDC lineage commitment via suppression of vascular smooth muscle differentiation through the up-regulation of *Tcf21*.

## 5. Epicardial Derived Cell Fate

Intense investigation has also focused on the fate of EPDCs. It is clear that EPDCs become vascular smooth muscle cells of the coronary arteries, as well as interstitial fibroblasts of the myocardium and endocardial cushions. Dettman *et al*. provided a convincing demonstration of this in 1998, utilizing LacZ stained quail epicardial cells grafted onto chick hearts. The cells were traced *in vivo*, and found to invade the sub-epicardial mesenchyme and myocardium, before forming the vascular smooth muscle of the coronary walls, as well as perivascular and intramyocardial fibroblasts. This study also demonstrated the ability to culture quail epicardial explants and induce epicardial EMT *in vitro* [67]. That same year, Gittenberger-de Groot replicated these data using similar techniques, and additionally demonstrated that EPDCs contribute to the valvular mesenchyme [68]. While these defining studies were conducted in avian species, more recent work done in mice has specifically demonstrated that EPDCs contribute to the definitive leaflets of the Atrioventricular Valves [55]. In addition to the valve leaflets, interstitial cells derived from EPDCs extend from the edges of the valves deep into the myocardium, thus contributing structural integrity to the heart via a contribution to the annulus fibrosis and the fibrous heart skeleton [52,69,70].

Outside of the vascular smooth muscle cell and fibroblast lineages, there is debate regarding the differentiation potential of EPDCs. Eralp *et al*. provided evidence for at least a supportive role for EPDCs in the development of Purkinje fibers and the electrical conduction system of the heart in the quail embryo [71]. Importantly, conflicting data have emerged regarding the ability of EPDCs to differentiate into cardiomyocytes. In 2008, Cai *et al*. and Zhou *et al*. independently used genetic lineage tracing to provide evidence that EPDCs differentiate into cardiomyocytes during murine heart development [72,73]. While these results are intriguing, the epicardial markers used in these studies are also expressed, though more rarely, in other populations of cells within the developing heart [74]. Well before these murine studies, Manner used cell tracing in quail-chick chimeras to demonstrate that EPDCs do not regularly contribute to the cardiomyocyte lineage in avian species [75].

Finally, there are conflicting data regarding the ability of EPDCs to differentiate into coronary endothelial cells. Lineage tracing studies carried out in quail-chick chimeras suggest that at least a subset of coronary endothelial cells originate from the epicardium, however, as discussed above, it is possible that endothelial lineage progenitor cells co-exist with VSMC and fibroblast progenitors in the PEO, thus representing a genetically distinct progenitor sub-population [76,77].

## 6. Function of the Epicardium Following Myocardial Injury

In recent years, the epicardium has garnered much attention as a potential source of cells for myocardial repair following injury. The reactivation of endogenous repair mechanisms would obviate several of the roadblocks inherent to cell-transplantation based attempts at myocardial repair, such as graft survival and immune rejection. The Zebrafish readily lends itself to the study of the epicardium in the setting of myocardial injury, given it’s unique ability to regenerate the myocardium following ischemic or mechanical insult. Following injury, the Zebrafish epicardium re-expresses *tbx18*, *wt1*, and the *Raldh2* ortholog *aldh1a2* [78,79,80,81]. Epicardial EMT is subsequently reactivated, resuling in epicardial cell proliferation and the generation of EPDCs and a subepicardial mesenchyme. Lineage tracing experiments and gene expression analysis have demonstrated that these EPDCs give rise to fibroblast-like cells and myofibroblasts that likely contribute to fibrosis, with no substantiated evidence suggesting that epicardial EMT serves to replenish the cardiomyocyte population within the infarct zone [11,82]. This is not to say that the reactivated epicardium does not play a non-cell autonomous role in the response to injury, as recent evidence suggests that the epicardium supports cardiomyocyte proliferation and migration following injury, particulary via the elaboration of RA and the cytokine Cxcl12a. Inhibition of the Cxcl12a receptor, Cxcr4, results in impaired myocardial regeneration [79,82,83,84]. Similary, following myocardial infarction in mice, there is a reactivation of embryonic epicardial gene expression, to include up-regulation of *Wt1, Tbx18, Raldh2*, and *Tcf21*. Intriguingly, c-kit^+^ EPDCs repopulate the sub-epicardial mesenchyme following injury, thus supporting the epicardium as a source of endogenous multipotency in the setting of ischemic infarction. *In vitro* work has likewise demonstrated that TGBβ-induced epicardial EMT results in EPDCs that express the c-kit antigen [85,86,87]. Katushka labeling carried out in 2010 by Gittenberger-de Groot *et al*. revealed that following MI in the mouse, the epicardial expression of *Wt1* is reactivated, with the production of new EPDCs that invade the underlying myocardium [85]. Concordant with a reactivation of embryonic epicardial gene expression and a resumption of epicardial EMT, multiple investigators have also demonstrated the up-regulation of downstream pathways important for the EMT process following experimentally induced MI. Wnt/β-catenin and Notch signaling, as well as SNAIL, and SLUG have all been demonstrated to increase in the epicardium and EPDCs following myocardial infarction [88,89,90].

The fate of EPDCs generated following murine MI appears to be similar that seen during heart development. In 2011, Zhou *et al*. demonstrated that following MI in the mouse, EPDCs expressed markers of fibroblast and vascular smooth muscle differentiation, but did not express cardiomyocyte or endothelial markers. Interestingly, the authors reported that though there was not a detectable cell autonomous contribution to revascularization, the EPDCs generated following infarction supported angiogenesis via the elaboration of paracrine mediators [90]. While these data do not support a direct differentiation of EPDCs into cardiomyocytes following injury alone, recent work has provided evidence that the fate of EPDCs can perhaps be manipulated exogenously to accomplish directed differentiation, as discussed below. Thus the available data currently suggest that in both the Zebrafish and the mouse, the epicardium retains the ability to reactivate epicardial EMT and contribute to the myocardial response to injury. This response appears to include a cell-autonomous contribution to the fibroblast and perivascular cell pools, and non-cell automous contributions to cardiomyocyte proliferation and migration, as well as angiogenesis in the infarct zone.

## 7. Summary and Future Directions

Over the last century, our view of the epicardium as a myocardial-derived barrier has been supplanted by an understanding of the epicardium as a highly functional epithelial monolayer that actively participates in heart development and the myocardial response to ischemic injury. Central to the function of the epicardium is the process of epicardial EMT, through which the epicardium makes cell-autonomous contributions to the developing and injured heart. We have begun to uncover the core inductive signals that govern the process of epicardial EMT, and the downstream pathways that are integral to epicardial cell transformation. The developmental plasticity of EPDCs has provided a basis for the investigation of epicardial EMT as a means of endogenous myocardial regeneration following ischemic infarction. Future regenerative therapies for ischemic myocardial injury will focus on the activation of endogenous cell sources in addition to exogenously delivered stem cells. In 2011, Smart *et al*. reported that intra-peritoneal injection of mice with thymosin β4 prior to experimentally induced myocardial infarction resulted in an induction of cardiomyocyte lineage selection by newly generated EPDCs [91]. These authors had previously demonstrated that thymosin β4 stimulated the differentiation of EPDCs into the endothelial lineage *in vitro* [92]. These data thus support a model in which exogenously administered compounds are employed to facilitate the directed differentiation of EPDCs that naturally occur in response to myocardial injury. Given that pre-infarction priming of the epicardium is not practical in a clinical sense, Zhou *et. al* tested the ability of thymosin β4 to induce cardiomyocyte lineage in EPDCs when injected after an experimentally induced MI. While these authors reported thickening of the epicardium and increased capillary density in injected animals, they did not report EPDCs transformation into cardiomyocytes or vascular endothelial cells [93].

Recent efforts have also focused on the transdifferentiation of resident cardiac fibroblasts into cardiomyocytes in the infarct zone [94,95,96]. Among the challenges encountered in the transdifferentiation of mature fibroblasts into induced cardiomyocytes (iCMs) is the complete epigenetic reprogramming required to push a mature cell into a completely different lineage, and future efforts will likely investigate the effect of exogenously administered compounds that exert an effect on the epigenetic landscape [91,95]. The larger effort to endogenously regenerate injured myocardium following infarction will thus employ both transdifferentiation techniques as well as the directed differentiation of EPDCs. By targeting cells with inherent multipotency (EPDCs), efforts aimed at the endogenous generation of cardiomyocytes following ischemic infarction may benefit from improved efficiency, secondary in part to the inherent epigenetic plasticity associated with this multipotency.
